# Exploration of Black Boxes of Supervised Machine Learning Models: A Demonstration on Development of Predictive Heart Risk Score

**DOI:** 10.1155/2022/5475313

**Published:** 2022-05-12

**Authors:** Mirza Rizwan Sajid, Arshad Ali Khan, Haitham M. Albar, Noryanti Muhammad, Waqas Sami, Syed Ahmad Chan Bukhari, Iram Wajahat

**Affiliations:** ^1^Department of Statistics, University of Gujrat, Hafiz Hayat Campus Jalalpur Road, Gujrat 50700, Pakistan; ^2^Faculty of Computing, Universiti Malaysia Pahang, Pekan 26600, Malaysia; ^3^Department of Surgery, College of Medicine, Majmaah University, Almajmaah 11952, Saudi Arabia; ^4^Centre of Excellence for Artificial Intelligence & Data Science, Department of Knowledge Management & Information Technology, Universiti Malaysia Pahang, Gambang 26300, Malaysia; ^5^Department of Community Medicine and Public Health, College of Medicine, Majmaah University, Almajmaah 11952, Saudi Arabia; ^6^Azra Naheed Medical College, Superior University, Lahore 54000, Pakistan; ^7^Division of Computer Science, Mathematics and Science, Collins College of Professional Studies, St. Johns University, New York, NY 11439, USA; ^8^Allied Institute of Medical Sciences, Gujranwala 52250, Pakistan

## Abstract

Machine learning (ML) often provides applicable high-performance models to facilitate decision-makers in various fields. However, this high performance is achieved at the expense of the interpretability of these models, which has been criticized by practitioners and has become a significant hindrance in their application. Therefore, in highly sensitive decisions, black boxes of ML models are not recommended. We proposed a novel methodology that uses complex supervised ML models and transforms them into simple, interpretable, transparent statistical models. This methodology is like stacking ensemble ML in which the best ML models are used as a base learner to compute relative feature weights. The index of these weights is further used as a single covariate in the simple logistic regression model to estimate the likelihood of an event. We tested this methodology on the primary dataset related to cardiovascular diseases (CVDs), the leading cause of mortalities in recent times. Therefore, early risk assessment is an important dimension that can potentially reduce the burden of CVDs and their related mortality through accurate but interpretable risk prediction models. We developed an artificial neural network and support vector machines based on ML models and transformed them into a simple statistical model and heart risk scores. These simplified models were found transparent, reliable, valid, interpretable, and approximate in predictions. The findings of this study suggest that complex supervised ML models can be efficiently transformed into simple statistical models that can also be validated.

## 1. Introduction

Machine learning (ML) models have gained significant importance in recent times due to their flexible nature to gauge complex phenomena accurately. However, this high performance often leads to the complexity of ML methods. Interestingly, the higher complexity of models brings higher accuracy, but it comes at the cost of the interpretability of the models [1, 2]. The trade-off between accuracy and interpretability of models has directed that generally, artificial neural network (ANN) models yield more accuracy but with the least interpretability [3]. On contrary, linear models are easy to interpret but with less accuracy. This issue of interpretability associated with ML models is termed a “black box.” It simply means that in ML processes, data go in, decisions come out, but the processes between input and output are opaque [4, 5]. However, the interpretability of models is as important as accuracy [6], especially in clinical decision-making [7].

In the domain of medical science, the understanding of how the models make predictions is the key to practitioners' trust in the model and its output [8]. Generally, the interpretable models are trustworthy because they are consistent with the prior knowledge and experience of practitioners. Additionally, the decision-makers can identify the unusual patterns within the data sets and explain them in a particular scenario. The black box nature of ML models has attained significant attention from experts in various fields. And technology giants such as Google, IBM, and Microsoft have been investigating the techniques and methods that can enhance the interpretability of these models [9]. The ML models focus on predictions and usually provide high accuracy, while the statistical models focus on drawing statistical inferences from these models [10]. Theoretically, this huge difference needs proper justifications when implementing ML models. Therefore, understanding the transparent process of developing ML models is always a key challenge for practitioners, especially when we want to prioritize them over traditional statistical models. Moreover, in the case of fatal diseases such as cardiovascular diseases (CVDs), it is and should be the fundamental requirement of clinical and public health experts while developing risk assessment models. Moreover, the need to have an accurate and validated heart risk score will have an impact on the estimation and prediction of the need for cardiac services and cardiac surgery in different settings with a low financial burden [11, 12].

A risk assessment or prediction model acts as a preventive strategy [13] and helps establish decision support systems [14]. Because of its simplicity and interpretability, logistic regression (LR) is a traditional statistical technique that is commonly used in developing such cardiovascular disease (CVD) related models [13]. However, the existing well-known risk prediction models fail, like the Framingham heart risk scores, which could not detect a large number of individuals accurately [15]. This failure increased the cost of treatment by identifying many individuals as members of the risky group who did not have the predicted level of risk [16, 17]. Therefore, researchers suggest that ML models can solve these accuracy-related issues [13, 18, 19]. In recent years, an extensive literature has been published regarding the utilization of ML models for CVD-related problems [15, 20–22]. These studies found that the performance of the complex ML models improved the prediction accuracy of the process compared to the existing statistical models. However, their “black box” nature is a potential hindrance in their utilization [1, 4, 7], and practitioners could not get an explanation for their prediction results and the role of features [23, 24]. The complexity and behavior of features within these opaque models pose a question about the interpretive abilities of the models. It motivates designing such methodologies that can help improve the interpretability of complex ML models with the minimum loss in performance. This study aims to propose and implement a methodology for transforming complex supervised ML-based risk prediction models into simple interpretable yet accurate risk prediction models. This proposed methodology was tested on the primary dataset, especially collected for the development of a predictive heart risk score using nonlaboratory features. The details of this data set have been provided in the subsequent Section.

## 2. Materials and Methods

**Figure 1:**
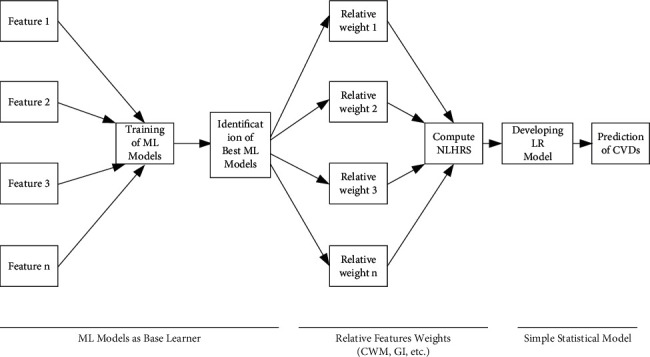
Proposed methodology for exploration of supervised ML models.

The proposed methodology is like a stacking ensemble that combines different simple and weak ML models for better performance [25]. However, in this study, we adopted it to improve ML models' interpretation and transparency and concluded with a simple LR model (see Figure 1). Mainly, it trained the ML models and derived the importance of the features through best-performed models. Furthermore, these extracted feature weights are used in the simple statistical model. The used ML models were from the class of artificial neural networks (ANN), support vector machines (SVM), and decision trees (DT). This methodology consists of a long process, and the same process has been followed for the derivation of novel nonlaboratory-based heart risk score (NLHRS) and their related risk prediction models. This NLHRS or heart risk score is specifically developed in this study. For easy implementation, in the development of this heart risk score, we only focused on such factors whose data are observational and can be collected without laboratory settings.

1. In the first step, supervised ML models that are DT, SVM with different kernels, and ANN were trained and implemented on the data set.
2. In the second step, the performance of these ML models was compared with that of an LR-based model. It is because LR is a commonly used statistical technique in the literature for prediction models. The ML models, which outperformed the LR model, will be continued in the next steps. This comparison is needed because weak ML models should be scrutinized to make this approach more efficient. Therefore, weak ML models were dropped for further processing. In this study, accuracy, sensitivity, specificity, area under the curve (AUC), root mean square error (RMSE), and kappa statistic were used to assess the performance of risk prediction models using tenfold cross-validation. We need to extract relative feature weights from the best-performed models in the next steps; therefore, this cross-validation was adopted because, in the training and testing approach, we can have different relative feature weights at various splits, that is, 80:20, 70:30, and 60:40, which can be problematic in further usage [26].
3. In the next step, relative feature weights were computed using a model-driven approach. It is also called a learner-based approach to extracting feature weights (see Figure 1). The best-performed ML models were employed to compute relative feature weights or importance. Input-hidden-output connection weight methodology (CWM) was used to compute relative feature weights in the finalized ANN model as suggested in the literature [27, 28]. DT uses information gain (IG) and the Gini index (GI) for assigning relative weights to features as discussed in the recent literature [26]. In addition, orthogonal vector coordinates orthogonal to the hyperplane are used by SVM to figure out the relative weights of features in the trained model. However, if the SVM model utilizes the radial basis function (RBF) kernel, then relative weights of features can be extracted through the recursive feature elimination (RFE) method as discussed in the literature [29].
4. Next, we transformed the original binary data set (explained in the next section) with newly computed relative feature weights. Initially, feature values in the data set were in the form of “0” and “1” and were labelled with “no” and “yes,” respectively. Yes = 1 indicated the exposure or presence of a particular feature. In the data transformation process, “1” was replaced with newly computed relative weights of features derived through the best ML models. Additionally, an additive approach was used to compute an index or score. However, the new score ranges from 0 to 100 while developing heart risk scores. In this study, these scores were denoted as ML-based NLHRS and were further used as input in the simple LR model for the development of NLHRS-based simple risk prediction models.
5. Finally, complete processes of performance assessment and validation were followed for these newly developed simple risk prediction models. The process of validation mainly evaluates the discrimination and calibration strengths of these simple risk prediction models are explained in the subsequent subsection. The valid NLHRS and associated risk prediction models can be used for screening of CVDs in the asymptomatic population.

### 2.1. Validation of NLHRS-Based Risk Prediction Models

In contrast to ML models, the proposed methodology will provide statistical models that can be validated through existing well-known methods. Therefore, in addition to the common performance matrices mentioned above, newly developed NLHRS-based risk prediction models were also validated. The basic purpose of this validation is to assess the discrimination and calibration capabilities of the models. Various statistical tests were used to evaluate the validity of these models. AUC, Brier score (BS), Spiegelhalter's Z-statistic, and Hosmer and Lemeshow (H) statistic were used for this purpose. AUC is used to assess the discriminative capability of the risk prediction model [30]. The relative BS provides an overview of the discriminative strength and calibration of the models [31]. In multiple models, Spiegelhalter's Z-statistic is used to make a comparison and help identify a better model [32]. Calibration is another important measure that assesses the agreement between observed and predicted outcomes [30]. H-statistic was used to measure the calibration of the model as suggested in the guidelines [33]. H-statistic is a goodness-of-fit (GoF) criteria in risk prediction models; therefore, its large value indicates the large difference between observed and predicted outcomes, leading to less calibrated models. For a good risk prediction model, the value of the H-statistic should be less than 20 [34]. In addition to H-statistic, calibration charts are also recommended to visualize the model's performance in various groups of probability [30].

### 2.2. Collection and Description of Data Set

In the development of NLHRS and its related risk prediction models, the same fifteen features were used as discussed in the literature [35, 36]. The features were gender, age in years, parental history of CVDs, dietary habits, physical activity, smoking, passive smoking, self-reported general stress, obesity, diabetes mellitus, and hypertension. Initially, except for age, all features were binary. However, we categorized age into two highly discriminative data-driven categories for scoring purposes as discussed in the literature [35, 36]. The QUEST algorithm, a type of DT, was used to derive the highly discriminative age categories, and the new binary feature is called “age groups.” This binary feature is utilized in the formulation of NLHRS and their corresponding risk prediction models. The data on these selected features were collected from the Punjab Institute of Cardiology (PIC), Pakistan, through a gender-matched case-control study. Male and female cases were matched with their corresponding male and female controls. Approval from the ethical review committee was also sought before the execution of the study. A sample of 460 subjects (with 1:1 matching) having ages >30 years were selected for this study in the duration of September 2018 to February 2019. Only those cases were selected who had their first CVD event (except congenital and rheumatic heart diseases) in their lifetime. The details of the data collection process and designs can be viewed in previous works [26, 37]. The empirical study was performed on Weka version 3.8 and Python 3.9.0.

## 3. Results

This study included 460 subjects with an equal distribution of cases and controls (230 cases and 230 controls). Among these subjects, 32.2% were females in both groups: cases and controls, because, it is already mentioned in the previous section that it is a gender-matched case-control study. The average age of subjects was 48 years with 11.31 years of standard deviation.

### 3.1. Implementation of Proposed Methodology for Computation of NLHRS

**Figure 2:**
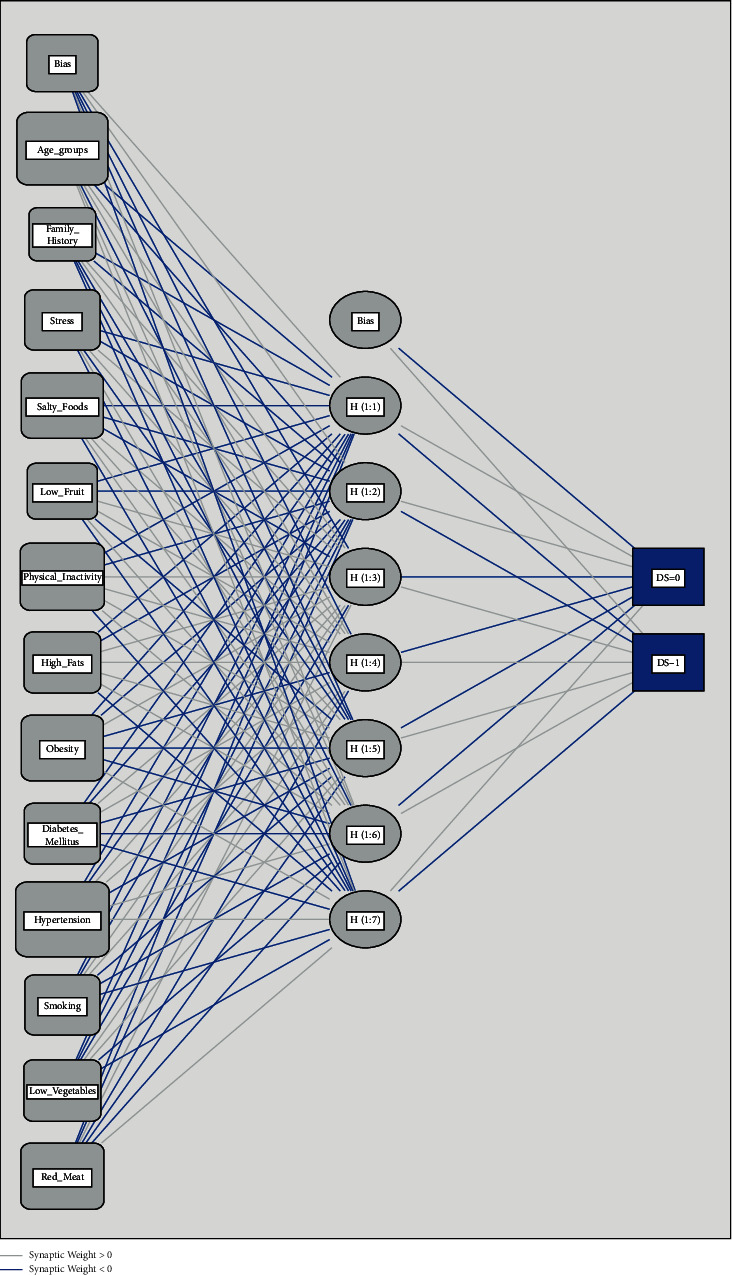
Illustration of finalized ANN model for prediction of disease status (DS).

Selected supervised ML models were implemented to train the models on the CVDs data set. These models were trained without gender as it was found insignificant due to the matching strategy and its insignificance can affect the performance of models. However, its confounding effect would be adjusted at a later stage. Passive smoking was an insignificant feature in the development of the LR model. It was found that the two ML models that are linear SVM and ANN outperformed LR and other ML models in the majority of the performance matrices (see Table 1). Therefore, it was excluded from further processing. We tried more than 50 configurations that include various numbers of hidden layers, number of nodes, and so on. However, the ANN model of a single hidden layer and 7 hidden nodes performed better than other ANN- and LR-based models (baseline model).  Figure 2 is the illustration of the ANN model and showed the complexity of the association between features and outcome variables. Various cost function values (*c*_*t*_) were implemented to optimize the linear SVM model, and *c*_*t*_ = 0.5 provided a good risk prediction model. It was found that ANN with a single hidden layer yielded the highest amount of accuracy (82.61%), specificity (0.848), AUC (0.881), and kappa statistic (0.653). Similarly, linear SVM also provided better performance matrices than the LR model. Therefore, linear SVM and ANN models were selected to evaluate model-driven relative feature weights. These two ML models fulfilled most performance criteria with good discrimination and calibration. These extracted feature weights were subsequently used to form the NLHRS and its corresponding risk prediction models. The NLHRS produced through the ANN model was named the “artificial neural network-based risk score” and is denoted by ANN-RS. Similarly, NLHRS produced through linear SVM is named support-vector-machine-based risk score (SVM-RS).

### 3.2. Extraction of Relative Feature Weights Using Best-Performed ML Models

The finalized relative feature weights were computed through CWM methodology in the ANN model and given in Table 2. The relative weights of features range from 5.138% to 11.944% in the overall prediction process (see Table 2). It is found that the feature “age groups” is crucial for the ANN-based risk prediction model since it receives the highest weight (11.944%). Hypertension is the second most important feature, with 10.527% relative weight in the ANN network. The least important feature identified by the ANN-based model with a relative weight of 5.138% is self-reported general stress.

Linear SVM is another ML best-performed model that was used for the computation of relative feature weights. Table 2 shows the relative feature weights computed through this model, ranging from 2.683% to 11.283%. Linear SVM-based feature weights identified that hypertension was the most discriminating feature for CVD status. Physical inactivity was the second most important feature in the linear SVM-based model. Contrary to the ANN-based model, the linear SVM ranked parental history of CVDs was the least discriminating feature for the model. Overall, it was found that both ML models have different patterns of features and different ranks. This is possibly due to the different nature of these two ML models.

### 3.3. Development of NLHRS-Based Risk Prediction Models and Their Performance Assessment

Now, the two types of NLHRS were represented as ANN-RS and SVM-RS and were used in the simple LR equation as proposed in methodology to formulate NLHRS-based risk prediction models (see Figure 1). ANN-RS and SVM-RS acted as a single covariate or prognostic index (PI) in two different LR equations. These PIs were denoted as PI_A_ and PI_S_ for ANN-RS and SVM-RS, respectively. PI_A_ and PI_S_ are the indexes that will be computed for an individual using the ANN- and SVM-based relative feature weights. For elaboration, an example is discussed here.

**Example 1 .**

For example, if a person is physically inactive, has a smoking history, consumes high salty foods, and has a history of hypertension and diabetes mellitus, then PI_*A*_ and PI_*S*_ will be computed as follows:

  PI_*A*_ = 8.024 (physical inactivity = 1) + 9.067 (smoking history = 1) + 6.128 (consumption of high salty foods = 1) + 10.527 (hypertension = 1) + 6.371 (diabetes mellitus = 1) = 40.12
  PI_*S*_ = 9.499 (physical inactivity = 1) + 7.887 (smoking history = 1) + 8.338 (consumption of high salty foods = 1) + 11.283 (hypertension = 1) + 7.961 (diabetes mellitus = 1) = 44.91

It was found that the regression coefficient of each type of PI was significant in their respective model. Furthermore, these equations were also adjusted for gender effects. These equations were referred to as NLHRS-based risk prediction model equations (see equations (1)–(4)). These simple LR-based equations with the PIs need to be validated before being used as a risk prediction model. Therefore, the predictive capability and validity of each NLHRS-based model were computed in the subsequent section. Equations (1) and (3) are the *z*_*i*_ (logits) of the ANN-RS and SVM-RS, respectively. However, equations (2) and (4) were used to estimate the probability of having CVDs based on ANN-RS and SVM-RS, respectively.

(1) $$\begin{array}{c} z_{i}=-5.659+0.107\left(\mathrm{Gender}\right)+0.160\left({\text{PI}}_{A}\right), \end{array}$$

(2) $$\begin{array}{c} P\left(\text{CVD}s=1|{\text{PI}}_{A}\right)=\;\frac{1}{1+e^{-z_{i}}}=\;\frac{1}{1+e^{-\left(-5.659+0.107\left(\mathrm{Gender}\right)+0.160\;\left({\text{PI}}_{A}\right)\right)}}, \end{array}$$

(3) $$\begin{array}{c} z_{i}=-6.131+0.415\left(\mathrm{Gender}\right)+0.174\;\left({\text{PI}}_{S}\right), \end{array}$$

(4) $$\begin{array}{c} P\left(\text{CVD}s=1|{\text{PI}}_{S}\right)=\;\frac{1}{1+e^{-z_{i}}}=\;\frac{1}{1+e^{-\left(-6.131+0.415\left(\mathrm{Gender}\right)+0.174\;\left({\text{PI}}_{S}\right)\right)}}. \end{array}$$

The performance of newly developed NLHRS-based risk prediction models was assessed and reported in Table 3. The results showed that the SVM-RS-based model had a relatively better accuracy of 83.50% with 0.888 AUC and the lowest RMSE value. The ANN-RS-based model provided 81% accuracy with 0.876 AUC. The accuracy of the ANN-RS-based risk prediction model was lower than the SVM-RS-based model. SVM-RS-based risk prediction model yielded improved findings than the original ML-based RPM provided in Table 1. However, the ANN-RS-based model provided a slightly compromised performance than the original ML-based risk prediction models. This slight loss of performance can be justified and possibly due to the small to medium size sample of the study. However, it shows that ML-based complex models are “wrapped up” within the newly developed simple NLHRS-based models.

### 3.4. Validation Process of NLHRS-Based Risk Prediction Models

In the medical sciences, the validity of the risk prediction model needs to undergo more rigorous evaluation. Therefore, there is a need for at least valid internal predictions of individuals from the underlying population [38]. This section specifically answers the question, “Are NLHRS-based risk prediction models valid?” Various statistical tests were used and reported here to evaluate the internal validity of NLHRS and its corresponding risk prediction models. The ideal values of these tests are provided in Table 4. The value of the BS score, which is close to 0, is ideal. It is found that the SVM-RS-based model has the lowest BS value of 0.115. The ANN-RS-based model provided a BS value of 0.142 that is far from the SVM-RS-based model. The lower value of BS indicates it has relatively well discriminated and calibrated NLHRS, but it also needs support from a test of significance. Therefore, Spiegelhalter's Z-statistic was used to assess the *H*_0_, which is similar to the H-statistic. The smaller value of Spiegelhalter's Z-statistic reflected a good overall calibration by the model. ANN-RS- and SVM-RS-based models provided –1.791 and –1.443 values of Spiegelhalter's Z-statistic, respectively, with *p*-value > 0.05. It means SVM-RS and ANN-RS provide well-calibrated NLHRS-based models.

**Figure 3:**
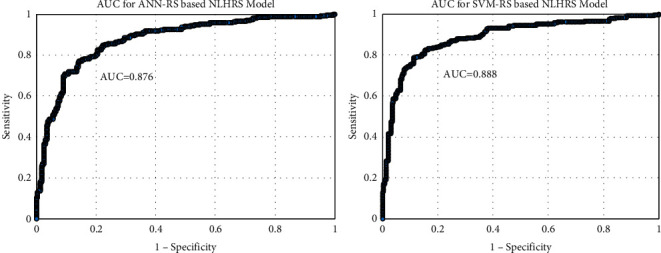
Discrimination strength of ANN-RS- and SVM-RS-based risk prediction models.

The SVM-RS-based risk prediction model achieved the highest value of AUC, but the ANN-RS-based model also depicts comparable findings with a point estimate of 0.876.  Figure 3 is the presentation of the discriminating capability of these two finalized models. These values indicate that the SVM-RS-based model had a better discriminating ability than the other model.

**Figure 4:**
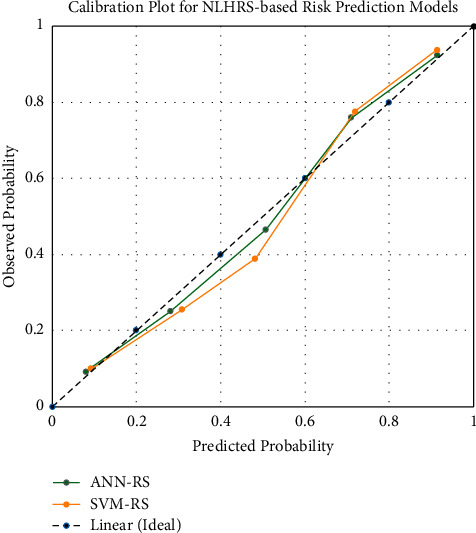
Calibration chart for NLHRS-based risk prediction models.

From Table 4, ANN-RS and SVM-RS showed good calibration as both models have H-statistic <20. Both NLHRS-based risk prediction models have acceptable levels of calibration. Furthermore, the required level of significance (>0.05) in both models was also achieved. It can also be observed from Figure 4 that ANN-RS-based risk prediction models showed better closeness with the ideal line as compared to SVM-RS models. Overall, the calibration plots graphically augmented the H-statistic findings and found the slight superiority of ANN-RS over the SVM-RS-based risk prediction model. However, irrespective of good calibration, the ANN-RS-based model had slightly compromised accuracy as discussed previously.

Overall, the performance and validity of NLHRS-based risk prediction models showed that both ANN-RS- and SVM-RS-based models yielded stable results, especially in the tests of significance such as H-statistic, Spiegelhalter's Z-statistic, and AUC. Except for calibration measurement, the SVM-RS-based risk prediction model was found to be more consistent in the performance and validation process. Therefore, these simple models with almost the same accuracy can reinforce the interpretability of initially developed complex ML models.

## 4. Discussion

The scope and application of complex ML models in decision support systems are continuously increasing due to their high performance. However, the usage of these effective but complex opaque models is a real challenge for practitioners. To help them, in this study, we proposed and implemented a methodology that efficiently transformed the complex supervised ML models into simple interpretable but approximately accurate statistical models. This methodology has been tested on a CVD-related data set. Two newly developed heart risk scores (ANN-RS and SVM-RS) and their corresponding models were found consistent in performance matrices, specifically with good discriminative and predictive strength. In addition to these matrices, the validation process of these models also augmented our proposed methodology. The small difference between the performance of complex ML-based risk prediction models and the transformed simple NLHRS-based risk prediction models is the key output of this proposed methodology.

Literature has shown an upward surge in the development and implementation of new ML algorithms. The performance of these algorithms is almost equal to humans or even surpasses them [1]. However, this constant improvement in the performance of these algorithms also increases their complexity, which attracted the attention of researchers [5, 39, 40]. The interpretability of models is an attractive characteristic, but the requirement for high performance in models cannot be undermined. Therefore, the balance of this trade-off is the ultimate requirement of researchers in recent works [1]. The literature has discussed that the simplicity of risk scores or models can come at the expense of performance, especially in terms of discrimination and calibration [41, 42, 43], and could increase the cost to the health system by providing wrong predictions [16]. This study provides a methodology that extracts the relative weights of features from best-performed complex ML models and utilizes them in a simple LR model to maintain the balance between interpretability and high performance of models. This unique combination of complex ML and simple LR models provides a hybrid approach of high performance and interpretability.

Our proposed methodology has provided models that are interpretable, can maintain performance, are transparent in the process, can be validated, and can be presented in statistical equations. Interestingly, these multiple characteristics are acquired at multiple stages of the proposed methodology. In the first stage, the actual input features were processed through complex ML models by considering possible problems of nonlinearity and interaction effects. This step provided higher accuracy than traditional statistical models such as LR. Using the relative feature weights approach, it tried to estimate the real weights utilized in the best ML models. This extraction of the relative weights of features highlighted the individual role of features in complex ML models and provided a transparent mechanism. The index of these relative feature weights acts as a single covariate in the LR model equation to form a simple statistical model. This step leads to simplicity, interpretability, and statistical form in the newly derived models. This simple LR model equation can easily be used due to its excellent interpretative capabilities, especially for medical scientists. Finally, parallel to existing LR-based models, newly developed models can also be validated along the same lines. These models also offer a continuous form of probability, but in simplicity, they are comparable to the existing semiquantitative models [44], which simply use an index of features for risk estimation of CVDs.

The newly developed heart risk scores, NLHRS, and their associated risk prediction models fulfill the majority of the criteria of a valid model, especially in terms of discrimination and calibration. Therefore, these two NLHRS-based risk prediction models can be used as screening tools for future purposes. In addition, this validated score can help predict the need for cardiac services in the different areas, especially in the absence of a good database. It also helps guide decision-makers to establish a cardiac unit or cardiac centre. The predictive performance and discrimination strength of the linear SVM-based NLHRS and its risk prediction model are relatively better than the ANN-RS-based model. Furthermore, the performance of linear SVM-based NLHRS and its related model has been improved from the original risk prediction models. However, the slightly compromised performance of ANN-based NLHRS and its model can be improved by using large data sets [45] and new methodologies for the extraction of feature weights. The findings of this study should be interpreted in the following limitations. Firstly, the proposed methodology has been tested on the CVDs data set that has been specifically collected for this purpose. Future researchers can test this methodology on other domains as well. Secondly, this study mainly focused on binary independent features. It is because the majority of risk factors of CVDs have binary nature. However, the proposed methodology can also be implemented on other types of data sets as well.

## 5. Conclusions

The conversion of complex ML models into simple but valid statistical models is the pertinent objective of the study. A novel methodology was adopted that uses the philosophy of stacking ensemble ML methods that combine different models for better performance. However, this complex process has been unfolded and yielded simple statistical models, which are interpretable, transparent, and valid. The proposed methodology had been tested on the primary data set and efficiently provided two valid heart risk scores, NLHRS, and their corresponding risk prediction models. Performance matrices generated by NLHRS-based models are approximately equal to the original complex ML-based risk prediction models. Therefore, it is suggested that this simple but transparent methodology can be used to develop interpretative models that use the efficient output of complex ML models as input. Furthermore, this validated risk score will have the potential for future use to predict cardiac diseases that will have an impact on clinical settings and future decision-making.

## Figures and Tables

**Table 1:** Performance assessment of ML models for the development of NLHRS.

Models	ANN		Linear SVM		RBF SVM		Random forest		LR	
Confusion matrix	Case	Control	Case	Control	Case	Control	Case	Control	Case	Control
Case	185	45	187	43	187	43	191	39	188	42
Control	35	195	46	184	50	180	56	174	50	180
Accuracy	82.61		80.65		79.80		79.30		80.00	
Sensitivity	0.791		0.813		0.813		0.830		0.817	
Specificity	0.848		0.800		0.783		0.757		0.783	
Kappa statistic	0.653		0.613		0.595		0.587		0.600	
AUC	0.883		0.881		0.870		0.857		0.873	
RMSE	0.365		0.372		0.379		0.411		0.380	
Number of criteria fulfilled	5/6		5/6		1/6		1/6		Baseline risk prediction model	

**Table 2:** Extraction of relative feature weights using ANN and linear SVM.

Features	Artificial neural network		Linear support vector machine	
	Sum of input feature contribution	Relative feature weights (%)	Original feature weights	Relative feature weights (%)
Age groups	0.836	11.944	1.085	8.828
Parental history of CVDs	0.401	5.735	0.330	2.683
Self-reported general stress	0.360	5.138	0.732	5.957
Consumption of high salty foods	0.429	6.128	1.024	8.338
Low fruit consumption	0.666	9.517	0.832	6.773
Physical inactivity	0.562	8.024	1.167	9.499
High fried foods/trans fats	0.404	5.770	0.857	6.976
Abdominal obesity	0.406	5.794	1.046	8.512
Diabetes mellitus	0.446	6.371	0.978	7.961
Hypertension	0.737	10.527	1.386	11.283
Smoking history	0.635	9.067	0.969	7.887
Low vegetables consumption	0.588	8.394	0.894	7.273
Red meat/poultry consumption	0.531	7.591	0.987	8.030
**Total**	**7.000**	**100.000**	**12.286**	**100.000**

**Table 3:** Performance assessment of NLHRS-based risk prediction models.

Models	ANN-RS		SVM-RS	
Confusion matrix	Case	Control	Case	Control
Case	190	40	190	40
Control	48	182	36	194
Accuracy	81.000		83.500	
Sensitivity	0.826		0.826	
Specificity	0.791		0.843	
Kappa statistic	0.620		0.670	
AUC	0.876		0.888	
RMSE	0.378		0.362	

**Table 4:** Validation of NLHRS-based risk prediction models.

Assessment	Test statistic/criteria	Ideal value	ANN-RS	SVM-RS
Overall discrimination and calibration	Brier mean probability score	0	0.142	0.115
Overall comparison of models	Spiegelhalter's Z-statistic (*p*-value)	0 (*p* > 0.05)	−1.791 (0.073)	−1.443 (0.150)
Calibration	H-statistic (*p*-value)	<20 (*p* > 0.05)	13.719 (0.089)	14.427 (0.071)
Discrimination	AUC	1	0.876	0.888

## Data Availability

The source of primary data used in this study is given in the manuscript. However, it is available on request to the corresponding author.
